# Effect of oxygen deficiency on the excited state kinetics of WO_3_ and implications for photocatalysis†
†Electronic supplementary information (ESI) available. See DOI: 10.1039/c9sc00693a


**DOI:** 10.1039/c9sc00693a

**Published:** 2019-05-09

**Authors:** Michael Sachs, Ji-Sang Park, Ernest Pastor, Andreas Kafizas, Anna A. Wilson, Laia Francàs, Sheraz Gul, Min Ling, Chris Blackman, Junko Yano, Aron Walsh, James R. Durrant

**Affiliations:** a Department of Chemistry , Imperial College London , London , SW7 2AZ , UK . Email: e.pastor11@imperial.ac.uk ; Email: j.durrant@imperial.ac.uk; b Department of Materials , Imperial College London , London , SW7 2AZ , UK; c Department of Materials Science and Engineering , Yonsei University , Seoul 03722 , Korea; d Molecular Biophysics and Integrated Bioimaging Division , Lawrence Berkeley National Laboratory , Berkeley , California 94720 , USA; e The Grantham Institute , Imperial College London , London , SW7 2AZ , UK; f Department of Chemistry , University College London , 20 Gordon Street , London , WC1H 0AJ , UK

## Abstract

Oxygen vacancies are widely used to tune the light absorption of semiconducting metal oxides, but a photophysical framework describing the impact of such point defects on the dynamics of photogenerated charges, and ultimately on catalysis, is still missing. We herein use WO_3_ as a model material and investigate the impact of significantly different degrees of oxygen deficiency on its excited state kinetics. For highly oxygen-deficient films, photoelectron spectroscopy shows an over 2 eV broad distribution of oxygen vacancy states within the bandgap which gives rise to extended visible light absorption. We examine the nature of this distribution using first-principles defect calculations and find that defects aggregate to form clusters rather than isolated vacancy sites. Using transient absorption spectroscopy, we observe trapping of photogenerated holes within 200 fs after excitation at high degrees of oxygen deficiency, which increases their lifetime at the expense of oxidative driving force. This loss in driving force limits the use of metal oxides with significant degrees of sub-stoichiometry to photocatalytic reactions that require low oxidation power such as pollutant degradation, and highlights the need to fine-tune vacancy state distributions for specific target reactions.

## Abstract 2

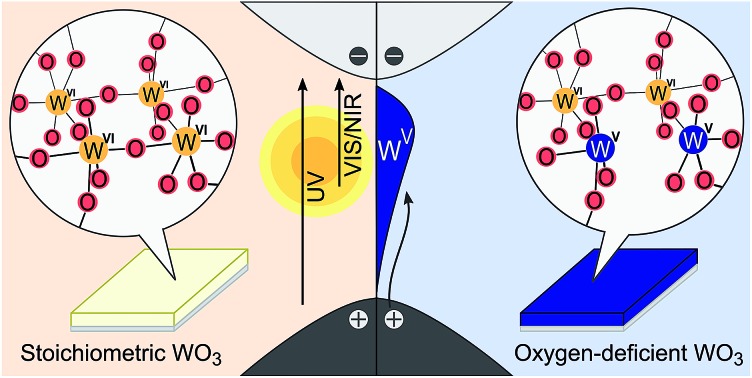
Using WO_3_ as a model material, we investigate how different oxygen vacancy concentrations affect trapping of photogenerated charges and photocatalytic reactions in metal oxides.

## Introduction

Due to the intermittency of sunlight there is growing interest in materials which can harness solar energy, not only for photovoltaic power generation, but also to drive the synthesis of sustainable fuels. Semiconducting metal oxides are attracting extensive interest as photocatalysts to produce such “solar fuels”, for example through photocatalytic splitting of water into molecular oxygen and hydrogen.^1^–^3^ Metal oxides are attractive for such applications due to their low cost, good chemical stability, and natural abundance. However, stable metal oxide photocatalysts often absorb poorly over the visible part of the solar spectrum. The fabrication of non-stoichiometric analogues is being widely explored as a potentially attractive route to overcome this problem and enhance their visible light absorption, in particular by introducing oxygen vacancies.^4^ However, despite their increased light absorption, the photocatalytic activity of such materials is typically found to decrease at higher levels of oxygen deficiency. To evaluate the practicability of this strategy, the impact of such modifications on the charge carrier dynamics that underpin photocatalytic efficiency requires closer investigation; in particular because studies investigating the impact of oxygen vacancies on charge carrier dynamics have been limited to date. To this end, we herein use one such metal oxide, WO_3_, both in its near-stoichiometric form and in a highly oxygen-deficient form, and investigate excited state processes upon irradiation of UV, visible, and near-infrared light.

While metal oxides are popular photocatalysts, they generally exhibit rapid recombination of photogenerated charges on picosecond to nanosecond timescales. These short charge carrier lifetimes lead to a severe mismatch with the timescales of desirable photocatalytic reactions such as water oxidation and proton reduction, which typically take place on the millisecond to second timescale. Carrier lifetimes are therefore a key limitation of this class of materials^5^ and chemical scavengers or applied electrical bias are usually employed to help overcome this constraint.^6^ In addition, many metal oxides have large bandgaps and thus often only absorb a small, high-energy fraction of the incident sunlight. This lack of visible light absorption is another major limitation of metal oxide based devices, as the high-energy UV light often needed to photoexcite these materials accounts for less than 5% of overall solar irradiance.^7^ To overcome this restraint, oxygen vacancies can be introduced into metal oxide lattices to extend the light absorption of these systems into the visible range. Numerous studies have explored this strategy and showed improved light absorption as well as enhanced photocatalytic activities for oxygen-deficient TiO_2_ 8–15 and other metal oxides such as WO_3_,^16^–^18^ ZnO,^7^,^19^,^20^ and SnO_2_.^21^ However, enhanced performance is typically only found in the case of moderate oxygen deficiency, whereas highly oxygen-deficient metal oxides with strong coloration counter-intuitively exhibit significantly lower photocatalytic activity than their stoichiometric analogues.^10^–^12^,^16^

WO_3_ is one of the prime members of the class of metal oxides and is widely used in photocatalysis and solar water-splitting devices.^22^,^23^ It is an n-type semiconductor with good charge carrier transport (electron mobility 10–40 cm^2^ V^–1^ S^–1^, hole mobility 3–10 cm^2^ V^–1^ S^–1^),^24^–^28^ is one of the few metal oxides which are stable against photocorrosion under acidic conditions (pH < 2),^29^,^30^ and exhibits particularly fast water oxidation kinetics.^31^ WO_3_ adopts a variety of different polymorphs, all of which are composed of a network of corner-sharing WO_6_ octahedra.^32^,^33^ Despite the popularity of WO_3_ for solar energy applications, only a few optical transient studies are available. Transient absorption studies reported the spectral fingerprints of photogenerated holes between 450 nm and 500 nm, whereas the absorption of photogenerated electrons was found at wavelengths higher than 750 nm.^34^ Electron trapping has been reported to occur on the order of 100 ps, which is significantly slower than in other metal oxides.^35^ In a recent transient photoconductivity study, mobile carriers were observed up to 600 ps for mesoporous WO_3_ films with average lifetimes of around 10 ps.^36^

We chose WO_3_ as a model system because it adopts sub-stoichiometric compositions more readily than other metal oxides. WO_3–*δ*_ is obtained through the release of oxygen from the crystal lattice, which causes the oxide to become reduced.^37^,^38^ Oxygen vacancies give rise to new electronic and optical levels within the bandgap of the semiconductor host. The creation of one doubly ionized oxygen vacancy 
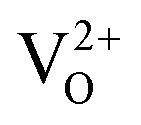
 leads to the formation of two excess electrons, which cause two one-electron reductions from W^VI^ to W^V^. In standard point defect notation, this corresponds to:
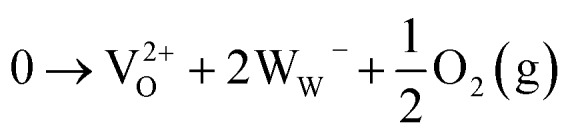


The reduced W_W_^–^ (*i.e.* W^V^) centres, which can be described as a small polaron due to strong local structure relaxation, may be in the vicinity of the oxygen vacancy (herein referred to as WVov) or far away in the stoichiometric crystal that gives rise to conduction band states (herein referred to as WVcb). W^V^ is an optically active d^1^ centre, which results in a distinct blue coloration. The concentration of reduced W centres, and thus the coloration intensity, can be modified in a number of different ways such as electrochemical, photochemical, and thermal stimuli.^39^,^40^ This facile change in coloration makes WO_3_ one of the leading materials for electrochromic applications and opens the door to tailoring the electronic structure of metal oxides to specific requirements.

Building on this coloration mechanism, we herein investigate the excited state dynamics of WO_3_ as a function of oxygen deficiency by monitoring the temporal evolution of photogenerated carriers *via* transient absorption spectroscopy (TAS). Knowledge about the interplay between reduced W centres in the process of visible light absorption makes WO_3_ particularly suitable for this study, as explicit assignments of transient signals to chemical species in metal oxides are usually challenging. We compare thin films of (i) regular monoclinic WO_3_ (m-WO_3_), and (ii) highly oxygen-deficient, blue WO_3_ (b-WO_3_). In particular, we compare the kinetics resulting from bandgap excitation using UV light to those resulting from sub-bandgap excitation using visible/near-infrared light. We find that the high degree of oxygen deficiency in b-WO_3_ considerably enhances the overall lifetime of charges generated by visible light absorption relative to m-WO_3_. However, in b-WO_3_, photogenerated holes quickly trap into sub-bandgap oxygen vacancy states, resulting in an energetic relaxation that compromises the efficiency of reactions that require high oxidative driving force. Quantum chemical calculations shed light on the nature of the optical levels introduced by oxygen vacancies, and further show that vacancy aggregates readily form in b-WO_3_, resulting in a broad distribution of sub-bandgap levels.

## Experimental

### Film preparation

b-WO_3_ thin films were deposited *via* aerosol assisted chemical vapor deposition based on a previously reported procedure.^41^ Following this procedure, 0.060 g W(CO)_6_ precursor was dissolved in a 2 : 1 mixture consisting of acetone (99%, Emplura) and methanol (99.5%, Emplura) with a total volume of 15 mL. Aerosols were generated from the resulting solution using an ultra-sonic humidifier (Liquifog, Johnson Matthey) operating at 2 MHz and were carried to the reactor using nitrogen gas (99.99%, BOC) at a flow rate of 400 sccm. The gas flow was regulated using a mass flow controller (MFC, Brooks). From these aerosols, thin films of b-WO_3_ were grown on quartz glass substrates held at 350 °C inside the reactor, after which heater and humidifier were switched off and the reactor was allowed to cool to room temperature while maintaining the nitrogen atmosphere.

m-WO_3_ thin films with near-stoichiometric composition were obtained by annealing b-WO_3_ thin films prepared as reported above in air. To this end, b-WO_3_ thin films were heated to 600 °C at a ramp rate of 4.8 °C min^–1^ and annealed at this temperature for 2 h. Subsequently, the annealed films were allowed to cool to room temperature inside the furnace.

### UV-NIR absorbance/reflectance and photoluminescence

Steady-state absorbance and reflectance data were acquired using a UV-vis spectrophotometer (Shimadzu UV-2600) equipped with a two-detector integrating sphere module (Shimadzu ISR-2600Plus). Photoluminescence spectra were acquired using a modular spectrofluorometer (Horiba Fluorolog-3).

### IPCE measurements

The incident photon to current efficiency (IPCE) was measured using a homemade PEC cell with a three-electrode set-up and WO_3_ films on an FTO coated glass substrate. The setup consisted of WO_3_ samples as photoanodes, a Pt mesh counter electrode and a Ag/AgCl/saturated-KCl reference electrode, submerged in a 0.1 M H_2_SO_4_ (pH 1) electrolyte. Measurements were taken under the illumination of a Xe lamp set to 75 W, and an applied potential of 1.23 V_RHE_, achieved using a potentiostat (Autolab, PGSTAT 12). The IPCE (%) was calculated using
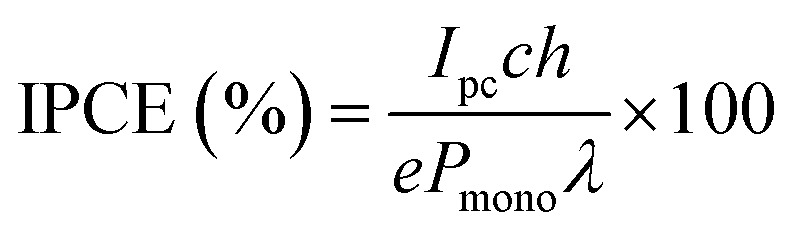
where *I*_pc_ is the measured photocurrent, *c* is the speed of light, *h* is Planck's constant, *e* is the elementary charge, *P*_mono_ is the monochromated light power and *λ* is the wavelength.

### Transient absorption spectroscopy (fs to ns timescale)

Transient absorption measurements on the fs to ns timescale were conducted using a regeneratively amplified Ti:sapphire laser system which has been described in detail elsewhere.^42^ The WO_3_ sample is probed both in its ground state and after excitation by a pump pulse of tuneable energy (320–1000 nm herein). By subtracting the absorbance in the ground state from that in the excited state, the absorbance difference Δ*A* as induced by the excitation pulse is obtained. The evolution of the excited state is then sampled by probing at different time delays with respect to the excitation event.

### Transient absorption spectroscopy (μs to s timescale)

Transient absorption data on the μs to s timescale was acquired using a home-built transient absorption spectrometer. The third harmonic output of a Nd:YAG laser (OPOTEK Opolette 355 II, 4–7 ns pulse width) was used for 355 nm excitation, and 600 nm excitation was achieved through an optical parametric oscillator. The laser output is transmitted to the sample *via* a liquid light guide. Excitation fluences were measured using a pyroelectric energy sensor (Ophir Photonics PE9). The monochromated output of a 100 W quartz halogen lamp (Bantham IL1) was used as a probe beam and was recorded by a Si photodiode detector (Hamamatsu S3071) after passing through the sample, with appropriate long pass filters positioned between sample and detector to attenuate scattered laser light. Data acquisitions were triggered by a photodiode (Thorlabs DET210) using scattered laser light. Data were recorded in a home-built LabVIEW-based software with an oscilloscope (Tektronix DPO 2012B) after amplification on the μs to ms timescale (Costronics 1999 amplifier) and simultaneously with a DAQ card (National Instruments, NI USB6211) on the ms to s timescale. The kinetic traces shown were typically obtained as an average of 80 individual excitation events with the subtraction of laser scatter. The acquired data was processed in OriginPro 2015/2017.

### X-ray diffraction (XRD)

X-ray diffraction (XRD) patterns were measured with a modified Bruker-Axs D8 diffractometer with parallel beam optics equipped with a PSD LinxEye silicon strip detector. The instrument uses a Cu source for X-ray generation (*V* = 40 kV, *I* = 30 mA) with Cu Kα_1_ (*λ* = 1.54056 Å) and Cu Kα_2_ radiation (*λ* = 1.54439 Å) emitted with an intensity ratio of 2 : 1. The incident beam was kept at 1° and the angular range of the patterns collected between 10 ≤ 2*θ*° ≤ 66 with a step size of 0.05°. Patterns were modelled using the Le Bail method with GSAS-EXPGUI software.^43^

### X-ray photoelectron spectroscopy (XPS)

X-ray photoelectron spectroscopy (XPS) was carried out using a Thermo Scientific K-Alpha instrument with monochromatic Al Kα source to identify the oxidation state and chemical constituents. Survey scans were collected over the 0 to 1400 eV binding energy range with 1 eV resolution and a pass energy of 200 eV. Higher resolution scans were collected in the binding energy regions of W (4f), O (1s), C (1s), Si (2p) and the valence band region (from –5 to 30 eV) with 0.1 eV resolution and a pass energy of 40 eV. An Ar-ion gun was used to etch the surface layers of samples to record a depth profile. Peaks were modelled using Casa XPS software with binding energies adjusted to adventitious carbon (284.5 eV).

### Hall effect

Room temperature Hall effect measurements were carried out on an Ecopia HMS-3000 in the van der Pauw configuration.^44^ Measurements were acquired at 0.58 T and a variable current of 0.1 mA to 1 nA on square-cut samples (∼1 × 1 cm). Silver paint (Agar Scientific) was used to form ohmic contacts, the integrity of which were tested prior to measurement.

### X-ray absorption spectroscopy (XAS)

X-ray absorption data at W L_III_-edge was collected on beamline 7-3 at Stanford Synchrotron Radiation Lightsource (SSRL) under standard ring conditions of 3.0 GeV and 500 mA current. A Si (220) double crystal monochromator was used for energy selection which was detuned to 50% of flux maximum at W L_III_-edge. The intensities of incident and transmitted X-ray beam were monitored using N_2_-filled ion chambers before the sample (*I*_0_) and after the sample (*I*_1_), respectively. The monochromator energy was calibrated with the first inflection point energy of a W foil spectrum (10.3 keV). The data was collected as fluorescence excitation spectra using a 30-element Ge solid-state detector (Canberra) and energy calibration for each spectrum was done using a monochromator crystal glitch in the *I*_0_ intensity relative to the absorption edge of W foil as no transmission data could be collected due to thick substrates. Data reduction of XAS spectra was performed using SamView (SixPack software, available at ; http://www.sams-xrays.com/sixpack). Athena program of Demeter software package (Demeter version 0.9.25, Ravel)^45^ was used to subtract the pre-edge and post-edge backgrounds from absorption spectra after which the spectra were normalized to the edge jump. EXAFS fitting was performed in *r*-space with Artemis software (Demeter version 0.9.25, Ravel) as previously described,^46^ using a passive electron reduction factor (*S*_0_^2^) of 0.85 (obtained from a fit of a W foil spectrum) and maintaining a total coordination number of *N*_tot_ = 6 whilst varying the coordination number of each shell (*N*) as shown in Table S1.†

### Density functional theory calculation

We performed hybrid density functional theory (DFT) calculations using the screened-hybrid exchange–correlation functional proposed by Heyd, Scuseria, and Ernzerhof (HSE06)^47^ and the projector-augmented wave (PAW)^48^ pseudo-potentials as implemented in the Vienna *Ab initio* Simulation Package (VASP). The plane-wave basis set^48^ was expanded up to 400 eV, and the atomic structures were optimized until the residual forces were less than 0.05 eV Å^–1^. We employed a 2 × 2 × 2 supercell containing 256 host atoms and used the *Γ* point for Brillouin zone integration. We used the SXDEFECTALIGN code to account for electrostatic potential alignment and finite-size effects in the formation energy of defects using the calculated low-frequency dielectric constants.^49^

## Results

Both types of WO_3_ thin films employed in this study exhibit a nano-needle structure with an overall film thickness of 150–200 nm as determined by scanning electron microscopy (Fig. S1†). Compared to the more oxygen deficient b-WO_3_, these nano-needles broaden during the annealing procedure and the film thickness reduces to *ca.* 150 nm. X-ray diffraction experiments (Fig. S2†) suggest monoclinic structures for both types of films, as is commonly observed for WO_3_ at room temperature.^16^,^24^,^50^ For b-WO_3_ two distinct peaks are observed, which have previously been attributed to the (010) and (020) reflections of the monoclinic W_17_O_47_ (WO_2.77_) structure with a [010] growth direction.^41^

**Fig. 1:**
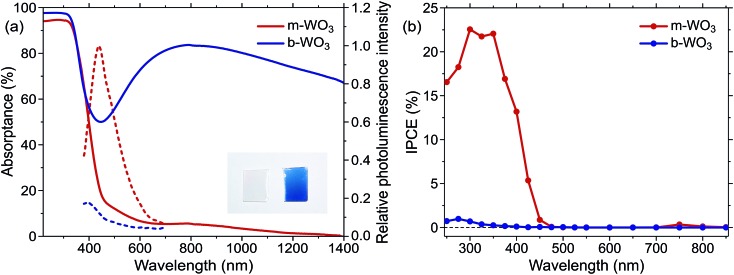
(a) Optical properties of the m-WO_3_ and b-WO_3_ thin films. UV-NIR absorptance spectra, calculated as 100-transmittance-reflectance (full lines), and photoluminescence emission spectra of m-WO_3_ and b-WO_3_ upon 355 nm excitation (dashed lines). The inset shows a photograph of the two types of films side by side. (b) Incident-photon-to-current conversion efficiency (IPCE) spectra for water oxidation at 1.23 V *vs.* RHE for m-WO_3_ and b-WO_3_ thin films.

The distinct optical properties of the two samples are evident from their striking difference in colour, as shown in Fig. 1a. In particular, b-WO_3_ exhibits a broad absorption feature throughout the visible and near-infrared region due to the absorption of reduced tungsten centres that are formed alongside oxygen vacancies. This polaron absorption is strongly suppressed in m-WO_3_. Indirect bandgap Tauc plots^51^ suggest that b-WO_3_ exhibits a slightly increased optical bandgap (2.90 ± 0.02 eV) compared to m-WO_3_ (2.80 ± 0.05 eV) (Fig. S3†). m-WO_3_ exhibits a sharp photoluminescence peak around 440 nm (2.82 eV), which we assign to the recombination of shallowly trapped charges based on the small energy difference with respect to the bandgap transition. In contrast, b-WO_3_ exhibits negligible photoluminescence (apart from that of the quartz glass substrate), which suggests that non-radiative recombination becomes more dominant as the concentration of oxygen vacancies increases. As shown in Fig. 1b, m-WO_3_ yields incident-photon-to-current conversion efficiencies (IPCEs) for water oxidation of up to 23% at 1.23 V *vs.* RHE, similar to the performance of analogously prepared WO_3_ films.^52^ Strikingly, b-WO_3_ films reach much lower IPCEs with maximum values below 1%. This dramatically reduced performance demonstrates that the extended visible light absorption of b-WO_3_ not only does not translate into visible light activity, but also compromises its activity in the UV region. In the following, we investigate the reasons for this pronounced difference in performance and explore strategies to control or bypass the associated losses.

**Fig. 2:**
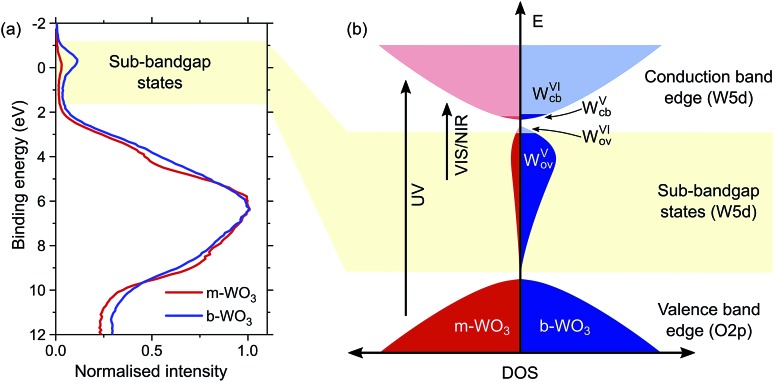
(a) XPS spectra of m-WO_3_ and b-WO_3_ at low binding energies. (b) Schematic of the electronic density of states in the bandgap region, which illustrates the broad distribution of sub-bandgap states due to defects and how thermal excitation leads to electrons in the conduction band, resulting in n-type conductivity. Transitions induced upon irradiation of UV and visible/near-infrared light are indicated exemplarily.

Quantum chemical calculations (discussed below) show that oxygen vacancies act as electron donors in WO_3_, converting the material to n-type (see Fig. S7†). The reduction of tungsten upon creation of oxygen vacancies was confirmed by W L_3_-edge X-ray absorption near-edge structure (XANES) spectroscopy measurements which show a shift of the absorption edge to lower energies as the oxygen vacancy concentration increases (Fig. S4†). Analysis of the extended X-ray absorption fine structure (EXAFS) reveals a higher heterogeneity of bond distances in more oxygen-deficient samples (Fig. S5 and Tables S1 and S2†). Consistent with this observation, calculations point to a substantial change in the local coordination environment upon formation of W^V^ polarons (*d*(W^VI^–O) = 1.89–1.95 Å, *d*(W^V^–O) = 1.75–1.92 Å). In addition, we employed X-ray photoelectron spectroscopy (XPS) to evaluate the composition of the surface of the film, which is of particular importance for interfacial reactions. Fig. 2a shows normalized spectra of our m-WO_3_ and b-WO_3_ films acquired at low binding energies, which reflect the density of occupied states in the bandgap region. The spectra consist of a broad peak at binding energies higher than 2 eV and a less intense, narrower peak at binding energies near 0 eV. These peaks are assigned to valence band states and WVov states, respectively. The signal ascribed to photoelectron emission from WVov states is considerably more intense for b-WO_3_ than for m-WO_3_, in agreement with the higher degree of oxygen deficiency in b-WO_3_ determined from XANES. Deconvolution of the W 4f photoelectron emission signal (Fig. S6†) reveals that 8% of all surface tungsten centres are in the W^V^ oxidation state for m-WO_3_, as opposed to 18% W^V^ for b-WO_3_. The difference in optical absorption is significantly larger than expected from this about twofold difference in W^V^ centres at the surface, which indicates the presence of a large number of bulk W^V^ centres in the case of b-WO_3_. The presence of additional bulk W^V^ centres in b-WO_3_ is in agreement with a larger intrinsic n-type carrier density, as determined from Hall effect measurements with ∼4 × 10^21^ cm^–3^ for b-WO_3_ and ∼5 × 10^16^ cm^–3^ for m-WO_3_. While the larger numbers of mobile carriers in b-WO_3_ result from thermal excitation of more sub-bandgap states close to the conduction band edge, the overall doping efficiency of b-WO_3_ (ratio of thermally excited states to overall number of states) can still be considered low due to the observed broad distribution of sub-band gap states.

**Fig. 3:**
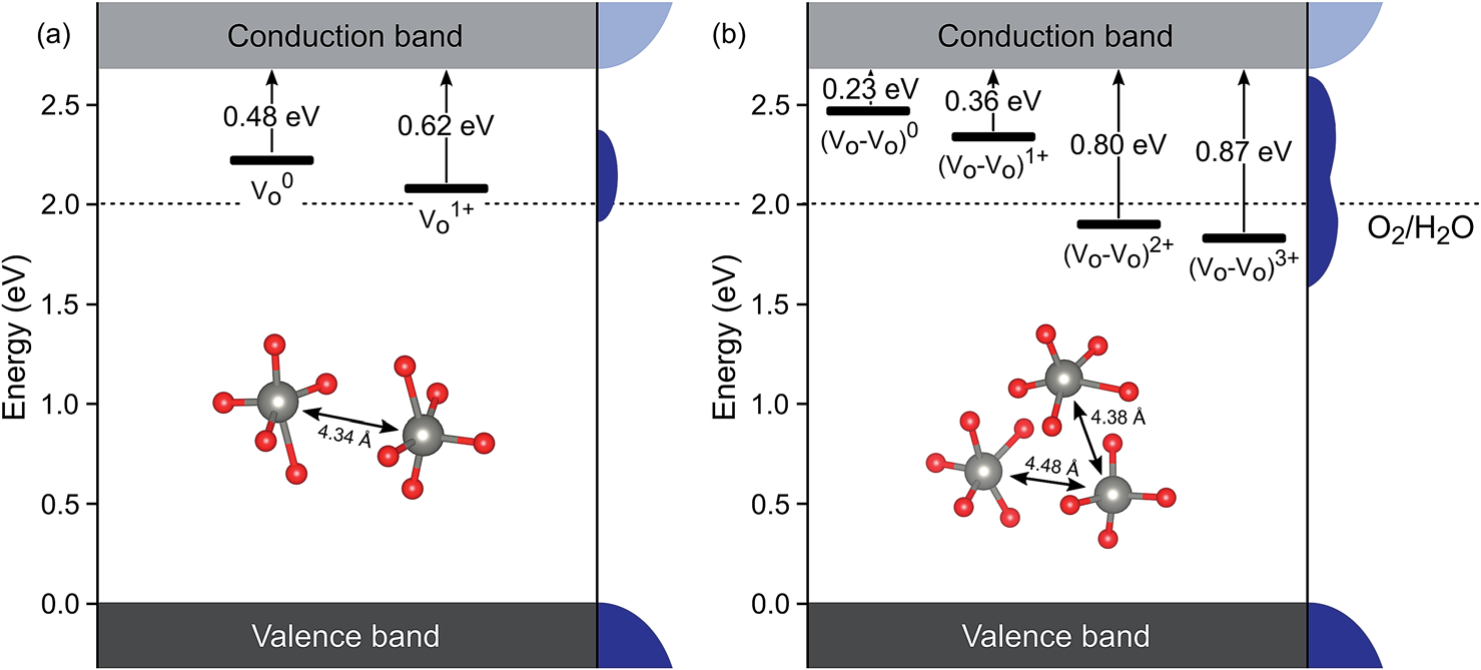
Defect ionisation levels of oxygen vacancies in different charge states calculated from hybrid density functional theory for (a) an isolated oxygen vacancy V_O_(*y*) and (b) a defect complex composed of two oxygen vacancies V_O_(*zy*). Energies are relative to the valence band edge. Densities of states are illustrated schematically at the right side of each panel. The insets show local atomic structures and the distance between the W atoms.

There is no consensus, from either experiment or theory, on the depth of the sub-bandgap levels associated with oxygen vacancies in WO_3_. We have performed first-principles calculations to investigate not only the isolated oxygen vacancy, but also defect complexes involving multiple vacancies, which may form in b-WO_3_. To examine whether the defect complexes are thermodynamically accessible, we first obtained the energy change upon forming a defect complex from two isolated oxygen vacancies. In standard point defect notation, the neutral oxygen vacancy can be written as 
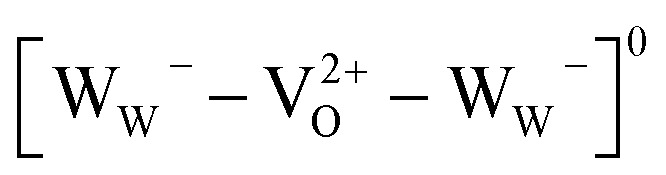
 and can be ionized to yield 
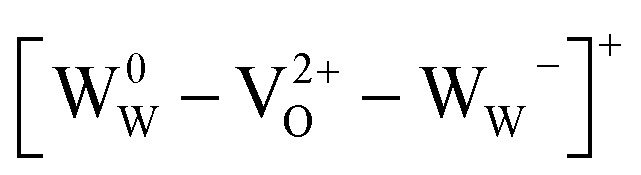
 and 
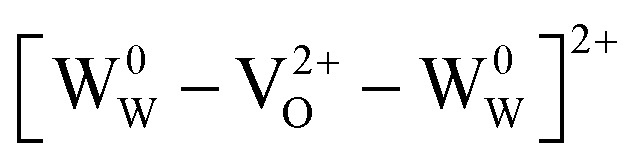
 (as discussed above, we note that W_W_^–^ in point defect notation corresponds to W^V^ in oxidation state notation). In monoclinic WO_3_, the three unit-cell axes are distinct and thus we removed an oxygen atom along the *x*, *y*, and *z* directions, which results in the formation of V_O_(*x*), V_O_(*y*), and V_O_(*z*) defects, respectively. Since the formation of V_O_(*x*) is less favourable (Fig. S7a†), we considered two defect complexes which we refer to as V_O_(*zy*) and V_O_(*zz*). The former defect complex, V_O_(*zy*), is composed of V_O_(*z*) and V_O_(*y*), while the latter V_O_(*zz*) is composed of two V_O_(*z*) defects. Formation of V_O_(*zz*) is endothermic (energy penalty of 1.8 eV per defect); however, the formation of V_O_(*zy*) is predicted to be exothermic (up to 0.4 eV) in n-type WO_3_ (Fig. S7b†). Fig. 3 shows energetic positions of oxygen vacancies in different charge states with respect to the valence and conduction band. Predicted states for an isolated V_O_(*y*) vacancy are located 0.48 and 0.62 eV below the conduction band. Upon V_O_(*zy*) complex formation, a wider range of charge states becomes accessible, giving rise to states between 0.23 and 0.87 eV below the conduction band. The corresponding configuration coordination diagrams for excitations between charge states of these oxygen vacancies are shown in Fig. S8 and S9.† These results demonstrate that defect–defect interactions can explain the broad distribution of oxygen vacancy states that is observed experimentally for b-WO_3_.

**Fig. 4:**
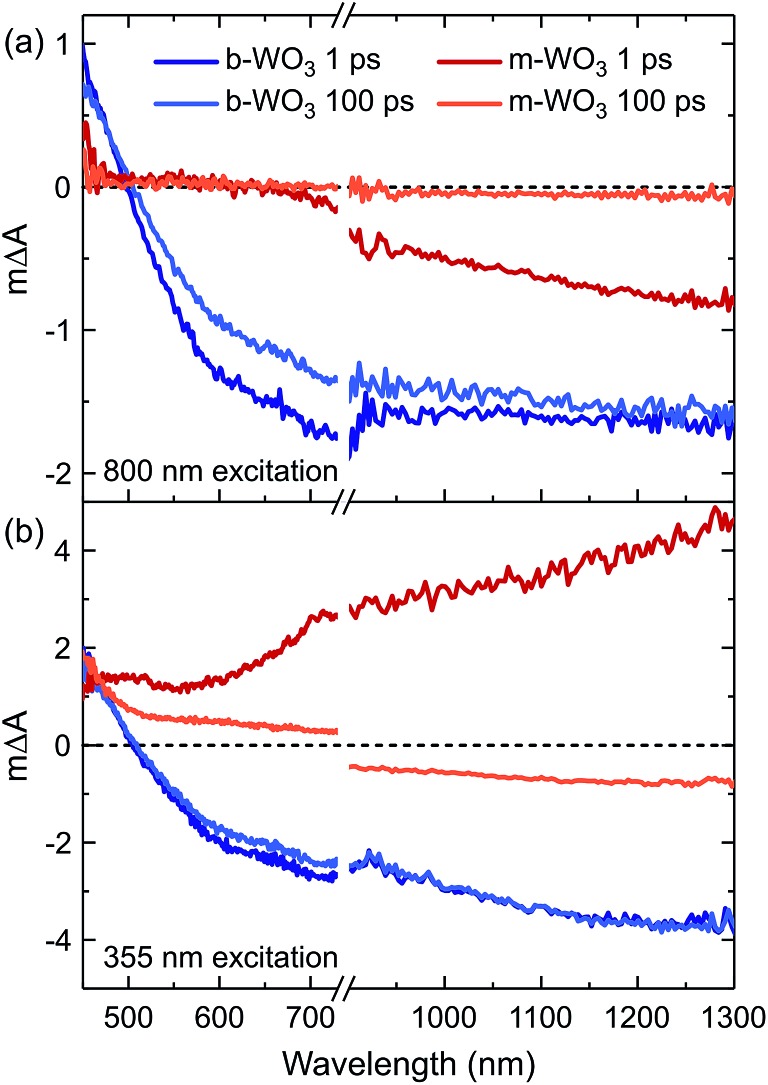
Transient absorption spectra of m-WO_3_ and b-WO_3_ for excitation wavelengths of (a) 800 nm and (b) 355 nm. For each sample and excitation wavelength, spectra probed at 1 ps and 100 ps after excitation are shown. All spectra were acquired at an excitation intensity of 0.26 mJ cm^–2^ under argon atmosphere.

We now turn to investigate how the differences in concentration and distribution of WVov states impact the charge carrier dynamics in the two WO_3_ samples. To this end, we perform TAS measurements on fs to ns timescales, probing absorption changes in the visible and near-infrared range upon pulsed photoexcitation. We use a range of different excitation wavelengths, which cover both bandgap excitation and the direct excitation of WVov states. Fig. 4 shows TA spectra of m-WO_3_ and b-WO_3_ thin films, obtained 1 ps and 100 ps after excitation at 355 nm and 800 nm. In both samples, a 355 nm excitation (Fig. 4b) induces a transition from the valence band to a conduction band state (forming WVcb). Excitation at 800 nm (Fig. 4a) promotes an electron from a vacancy centre (WVov) to the bulk conduction band (forming WVcb). In these data, a positive Δ*A* reflects the absorption of a state that is newly generated upon photoexcitation (for example generation of WVcb). In contrast, a negative Δ*A* results from the photoinduced depopulation of an absorbing ground state (for example depopulation of WVov), which leads to an overall lower absorption in the excited state. In this way, such transient spectra can be used to identify photogenerated species at a given time after the irradiation of a light pulse with defined energy.

All transient spectra show broad photoinduced absorption features. In previous studies of WO_3_, a positive photoinduced TA between 450 nm and 500 nm was mostly attributed to holes, whereas electrons (*e.g.* WVcb) dominate the spectrum above 750 nm.^34^ We start by examining m-WO_3_ at sub-bandgap excitation: excitation using visible/near-infrared light ionizes oxygen vacancies. This ionization process corresponds to the conversion of WVov (excess electron trapped at defect) to WVcb (excess electron in the bulk conduction band). In this scenario, the only viable relaxation pathway is the regeneration of WVov states *via* re-trapping of the photoexcited electron.

As shown in Fig. 4a at 1 ps after excitation at 800 nm, a bleach (Δ*A* < 0) is observed towards the near-infrared region which almost fully decays within 100 ps, indicating that most photoexcited electrons re-trap within this timescale. The dominant character of this bleach indicates that the negative signal associated with the loss of WVov contributes more strongly to the overall spectrum than the positive signal from simultaneously created WVcb, which can be ascribed to a higher absorption coefficient of the respective WVov transition. In contrast, when exciting m-WO_3_ above its bandgap using 355 nm light, a positive absorption feature is found after 1 ps throughout the probed spectral range (Fig. 4b) and can be assigned predominantly to the photoinduced absorption of valence band holes below 500 nm and WVcb conduction band electrons above 700 nm. After 100 ps, this positive absorption is partially retained in the visible range, but a negative signal dominates the near-infrared range. Interestingly, this negative feature resembles that observed after 1 ps upon 800 nm excitation (Fig. 4a). This spectral similarity suggests that WVIov states are also generated through excitation above bandgap, but at later times. We associate this behaviour with the trapping of valence band holes by WVov. This trapping process is therefore analogous to the creation of WVIov upon sub-bandgap excitation of WVov. We note that this bleach is not observed at microsecond times for m-WO_3_ (Fig. S10†), which suggests that holes trapped at oxygen vacancies recombine with conduction band electrons on the nanosecond timescale. A small positive signal is retained below 500 nm, which can be ascribed to a small residual population of valence band holes.

In the case of the highly oxygen deficient b-WO_3_, sub-bandgap excitation at 800 nm yields a distinct spectral signature consisting of a positive TA below and a negative TA above 500 nm, both at 1 ps and 100 ps time delay (Fig. 4a). The negative change in absorbance occurs over the same spectral range as the WVov absorption that gives the film its blue coloration (Fig. 1), indicating that this negative feature originates from the depopulation of these states. Unlike in the case of m-WO_3_ where the transient spectrum depends strongly on excitation wavelength, the same spectral signature is observed upon bandgap excitation using 355 nm light (Fig. 4b). Therefore, we conclude that WVIov are generated in b-WO_3_ within 1 ps after excitation for both bandgap and sub-bandgap excitation. Transient absorption experiments on longer timescales show that this negative feature persists up to several milliseconds and follows an identical decay for the two different excitation wavelengths, further emphasizing that both bandgap and sub-bandgap excitation result in the creation of WVIov (Fig. S11†).

**Fig. 5:**
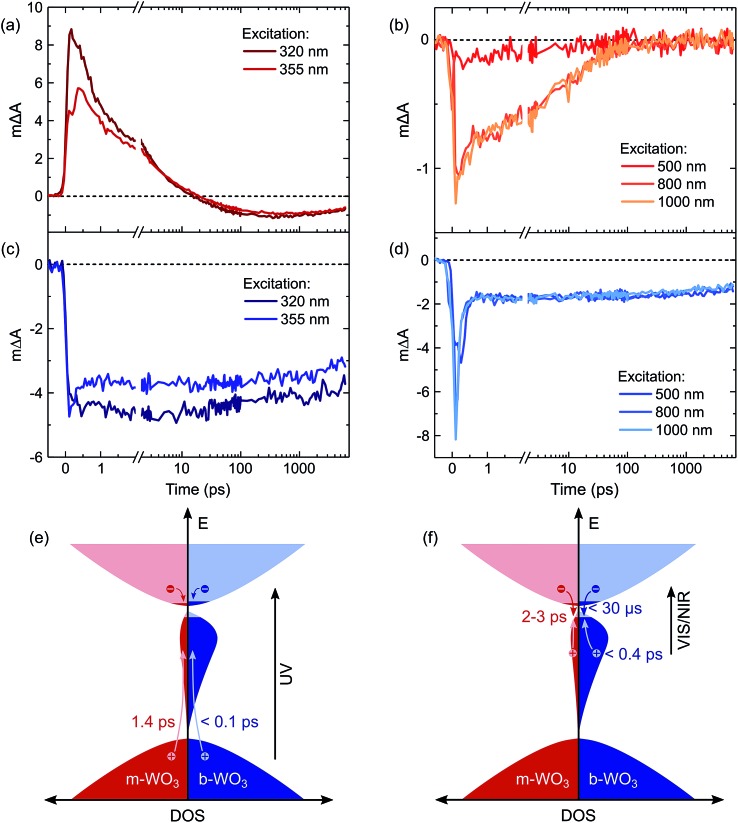
Transient absorption decay kinetics probed at 1200 nm for (a) bandgap excitation of m-WO_3_, (b) sub-bandgap excitation of m-WO_3_, (c) bandgap excitation of b-WO_3_, and (d) sub-bandgap excitation of b-WO_3_. All kinetics were acquired at an excitation intensity of 0.26 mJ cm^–2^ under argon atmosphere. A model illustrates the corresponding photoinduced processes for bandgap excitation (e) and sub-bandgap excitation (f) of both m-WO_3_ and b-WO_3_. The indicated times are decay half-times for the respective processes. For simplicity, we show WVcb states (electrons away from a vacancy) only in the conduction band; however, we note that polaron formation through self-trapping of conduction band electrons could also lead to localization away from a vacancy at energies within the bandgap.

For a more detailed investigation of the photoinduced processes, we inspect the charge carrier kinetics for a broader range of excitation wavelengths. Fig. 5 shows kinetics probed at 1200 nm for bandgap excitation (Fig. 5a and c) and sub-bandgap excitation (Fig. 5b and d). Upon bandgap excitation of m-WO_3_, a positive excited state absorption evolves into a bleach with a half-time of 1.4 ps for 355 nm excitation, reaching its maximum negative amplitude around 400 ps (Fig. 5a). As stated above, such evolution suggests that the initially positive transient absorption from WVcb is increasingly dominated by the generation of WVIov through trapping of valence band holes. In contrast, sub-bandgap excitation directly generates WVIov and thus gives rise to an immediate bleach (Fig. 5b). This bleach undergoes a stretched exponential decay (Δ*A*(*t*) ∝ exp(–(*t*/*τ*))^*b*^ with *b* varying 0.19–0.34) which implies that photogenerated carriers are characterized by a distribution of different lifetimes,^36^,^53^ as can be expected for a distribution of trap states at different energies within the bandgap.

The decay behaviour of the more oxygen deficient b-WO_3_ is substantially different. Bandgap excitation gives rise to an immediate and long-lived bleach, in stark contrast to m-WO_3_ where bleaching only occurs from 20 ps onwards. We therefore conclude that trapping of valence band holes in WVov states takes place within our time resolution (<200 fs) in b-WO_3_. This fast hole trapping is consistent with the significantly higher concentration of WVov present within the bandgap of b-WO_3_. This fast trapping process explains the near-complete absence of visible photoluminescence from b-WO_3_ (Fig. 1) as holes do not reside in the valence band long enough to undergo radiative recombination. Upon sub-bandgap excitation, a similar long-lived bleach is observed, but with an additional fast decay phase below 0.8 ps (Fig. 5d), which is discussed further below. Interestingly, the long-lived negative components observed for both bandgap and sub-bandgap excitation of b-WO_3_ persist until microsecond to early millisecond time scales and both follow indistinguishable power law decay (Δ*A*(*t*) ∝ *t*^–*a*^ with *a* = 0.51) at these longer times (Fig. S11†), which suggests highly dispersive recombination in the presence of trap states.^54^ Such identical decay behaviour provides further evidence that the transient signal obtained in b-WO_3_ can be assigned to trapped holes in the form of WVIov states in the case of both bandgap and sub-bandgap excitation. These trapped holes were found to be unreactive towards high concentrations of commonly used hole scavengers such as methanol, Na_2_SO_3_, and triethanolamine (Fig. S12†), suggesting that they are either thermodynamically unable to oxidize these substrates or spatially located too far from the surface to interact with them.

## Discussion

Herein, we report an analysis of the charge carrier dynamics in near-stoichiometric and strongly oxygen-deficient tungsten oxide thin films (m-WO_3_ and b-WO_3_, respectively). Fig. 5e and f summarize our key findings. For both films, our transient data show that following bandgap excitation, photogenerated valence band holes rapidly trap into occupied oxygen vacancy states within the bandgap (denoted WVov herein). For m-WO_3_ this hole trapping process is highly dispersive, with a decay half-time of *ca.* 1 ps but extending out to 200 ps. For b-WO_3_ hole trapping occurs within our time resolution (<200 fs), consistent with the higher density of WVov states in these films as determined from UV-vis, XPS, and XANES measurements. In contrast, sub-bandgap excitation results in the direct generation of holes in sub-bandgap states which recombine with a decay half-time of 1–2 ps in m-WO_3_, but persist up to milliseconds in b-WO_3_. Hall effect measurements demonstrate that the concentration of intrinsic mobile carriers in b-WO_3_ is increased by ∼5 orders of magnitude compared to m-WO_3_. Nevertheless, the broad distribution of occupied states in the bandgap, as determined by XPS and first principles defect calculations, suggests an overall low doping efficiency as most sub-bandgap states are too far from the conduction band for thermal excitation and thus remain occupied. Previous theoretical studies suggested that single oxygen vacancy defects are shallow in WO_3_, however, our calculations imply that oxygen vacancies form defect complexes and result in deeper donor level and a wider range of defect states in the bandgap.

In a general picture, occupied sub-bandgap oxygen vacancy states (WVov) act as hole traps whereas unoccupied ones (WVIov) can trap electrons. The number of WVov states is much higher than that of WVIov states due to a low electronic doping efficiency, and the trapping of photogenerated holes is thus the dominant process. Because the density of WVov states extends towards the conduction band edge, this trapping process results in a large energy loss (deep trapping) as demonstrated by the energy difference between valence band and calculated oxygen vacancy energies in Fig. 3. For instance, a relaxation from the valence band edge to high-lying WVov states as determined by XPS/DFT would correspond to an energy loss of ∼2 eV or more. This hole relaxation can be observed upon sub-bandgap excitation of b-WO_3_ which results in a fast decay component on the femtosecond timescale (Fig. 5d). Such behaviour is characteristic of fast carrier relaxation through a high density of states as is usually observed for metals and degenerate semiconductors,^55^–^57^ and has recently been described for substoichiometric WO_3_.^58^ We note this decay could also be associated with geminate recombination losses resulting from the more localized nature of the optically excited oxygen vacancy states. The constant signal amplitude following this fast decay can be correlated to holes in sub-bandgap oxygen vacancy states close to the conduction band. The long lifetime of these holes can be related to their deeply trapped character. The different coordination environments that give rise to a broad WVov distribution in our DFT calculations suggest that carriers might be trapped at different sites within the material, thus slowing down recombination. In contrast, sub-bandgap excitation of m-WO_3_ results in a stretched exponential decay (Fig. 5b) which suggests that holes recombine from various energy levels within the bandgap, indicating that such energetic relaxation of photogenerated holes is less pronounced than in b-WO_3_. As opposed to the trapping of photogenerated holes, trapping of photogenerated electrons into the rather low density of WVIov states results in a relatively small energy loss (shallow trapping). Our observations are consistent with reports on α-Fe_2_O_3_ photoelectrodes where analogous hole trapping into occupied oxygen vacancy states was found to be prominent in the absence of a space charge layer.^54^ Conversely, optical excitation within a space charge layer generated by strong anodic potentials gave rise to electron trapping into now unoccupied oxygen vacancy states, resulting in the accumulation of α-Fe_2_O_3_ holes with a sufficient lifetime for water oxidation at the photoelectrode surface.^54^,^59^

The highest photocatalytic activities are typically found for moderately oxygen-deficient films which do not have excessive numbers of sub-bandgap states and therefore do not exhibit intense coloration. In agreement with our IPCE data (Fig. 1b), little to no activity has been found for different metal oxides when irradiating light with an energy corresponding to the absorption features that arise from sub-bandgap oxygen vacancy states.^8^,^10^–^12^,^16^,^20^,^60^ Instead, the observed performance enhancement for moderately oxygen-deficient oxides is attributed to a more efficient conversion of high energy photons.^61^ This improved conversion efficiency can be linked to improved conductivity as a direct result of the oxygen vacancy induced increased doping density,^62^ as well as enhanced band bending close to the semiconductor interface^50^ where most high energy photons are absorbed. More detailed investigations show that such improved conductivity can be related to suppressed recombination on slow timescales.^59^,^63^ The high activity at moderate oxygen deficiency suggests that a balance between a sufficiently high doping density and a sufficiently low number of WVov states is necessary to achieve good performance. With these two quantities being closely related, insights from our transient data points to a trade-off between a favourable enhancement in conductivity through higher doping densities and an unfavourable decrease in driving force through a higher number of hole trap states. Once the number of WVov states becomes too high, such losses in driving force severely compromise the efficiency of oxidation reactions that require oxidative power, despite the increase in carrier lifetime. We note that similar issues occur in carbon nitride photocatalysts, where charge carrier relaxation into trap states results in slower recombination times but at the expense of a loss of energy to drive photocatalytic reactions.^64^ Based on these insights, it seems unlikely that metal oxides with excessive oxygen vacancy densities can achieve higher activities for demanding oxidation reactions such as water oxidation than their more stoichiometric analogues. However, the longer hole lifetime might be advantageous for more facile reactions with less positive oxidation potentials such as pollutant degradations. On the other hand, electron trapping can be expected to result in a less substantial loss in reductive driving force due to the absence of deep electron traps, which implies potential for applications such as CO_2_ reduction.^65^ Overall, it would be desirable to develop ways to engineer oxygen vacancies in order to fine-tune the energetic positions of the formed sub-bandgap states and thus control losses in driving force.

## Summary

This study provides a direct comparison of charge carrier dynamics in WO_3_ with low and high oxygen vacancy densities, referred to as m-WO_3_ and b-WO_3_, respectively. Oxygen vacancy formation introduces sub-bandgap states, herein denoted WVov and WVIov depending on their occupation. First-principles defect theory shows that defect clustering can produce broad distributions of sub-bandgap states such as that observed in our b-WO_3_ film. As most oxygen vacancy states lie deep within the bandgap of this n-type semiconductor, they will remain largely occupied (WVov). Thermal excitation to the conduction band (producing WVIov) can only occur for a small fraction of oxygen vacancy states close to the band edge, meaning that the material is characterized by a low electronic doping efficiency. The occupied WVov states act as trapping sites for photogenerated valence band holes. Upon bandgap excitation of the near-stoichiometric m-WO_3_, trapping of valence band holes into these WVov states occurs on a time scale of up to 200 ps, with a decay half-time of 2–3 ps. In contrast, this trapping process occurs within 200 fs in the highly oxygen-deficient b-WO_3_ due to the higher density of WVov states, leading to near-complete photoluminescence quenching. While this fast hole trapping in b-WO_3_ leads to a significantly enhanced lifetime of charges generated by visible/near-infrared light, it also results in a significant loss of oxidative driving force. For highly oxygen-deficient WO_3_ films, this loss in driving force compromises the photocatalytic activity for demanding oxidation reactions such as water oxidation and explains why the best photoelectrochemical water oxidation performance is typically observed for metal oxide films with moderate degrees of oxygen deficiency. A moderate oxygen vacancy density can result in a higher intrinsic carrier density, improving electrode conductivity and allowing the formation of a well-defined space charge layer. Despite the rapid relaxation of photogenerated holes in films with high oxygen vacancy densities, the enhanced lifetime of the photogenerated carriers could be beneficial for reactions with less positive oxidation potentials such as pollutant degradation or for suitable reduction reactions.

The results presented herein provide a fundamental understanding of why the energetic distribution of sub-bandgap states, and not just that of valence and conduction band states, needs to be adapted to the redox potential of a targeted photocatalytic reaction.

## Conflicts of interest

There are no conflicts to declare.

## Supplementary Material

Supplementary information
