# Supplementary material for: Effect of oxygen deficiency on the excited state kinetics of WO3 and implications for photocatalysis
Source: Chem Sci. 2019 May 9;10(22):5667–77. doi: 10.1039/c9sc00693a (PMC6563783; doi:10.1039/c9sc00693a)
Supplement: Supplementary file 1 [file SC-010-C9SC00693A-s001.pdf]

Electronic Supplementary Material (ESI) for Chemical Science.
This journal is © The Royal Society of Chemistry 2019

Supporting Information

Effect of oxygen deficiency on the excited state kinetics of WO_3 and implications for photocatalysis

Michael Sachs,[a] Ji-Sang Park,[bc] Ernest Pastor,*[a] Andreas Kafizas,[ae] Anna A. Wilson,[a] Laia Francàs,[a] Sheraz Gul,[d] Min Ling,[f] Chris Blackman,[f] Junko Yano,[d] Aron Walsh,[bc] and James R. Durrant*[a]

[a] Department of Chemistry, Imperial College London, London, SW7 2AZ, U.K.

[b] Department of Materials, Imperial College London, London, SW7 2AZ, U.K.

[c] Department of Materials Science and Engineering, Yonsei University, Seoul 03722, Korea

[d] Molecular Biophysics and Integrated Bioimaging Division, Lawrence Berkeley National Laboratory, Berkeley, California 94720, U.S.A.

[e] The Grantham Institute, Imperial College London, London, SW7 2AZ, U.K.

[f] Department of Chemistry, University College London, 20 Gordon Street, London, WC1H 0AJ, U.K.

E-mail: e.pastor11@imperial.ac.uk, j.durrant@imperial.ac.uk

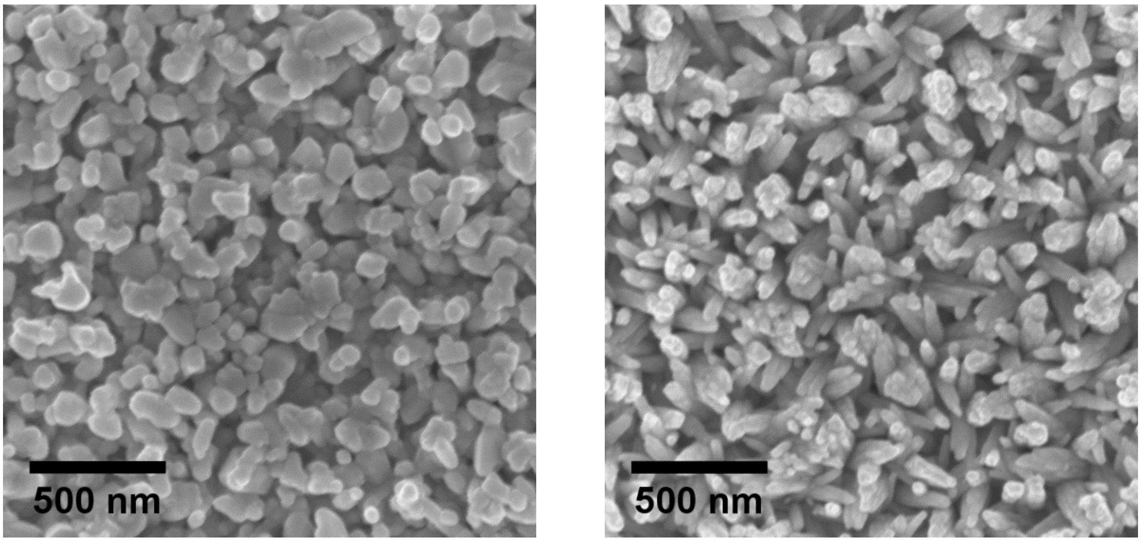

Figure S1. Scanning electron micrographs of m-WO_3 (left) and b-WO_3 (right) thin films on quartz glass substrates.

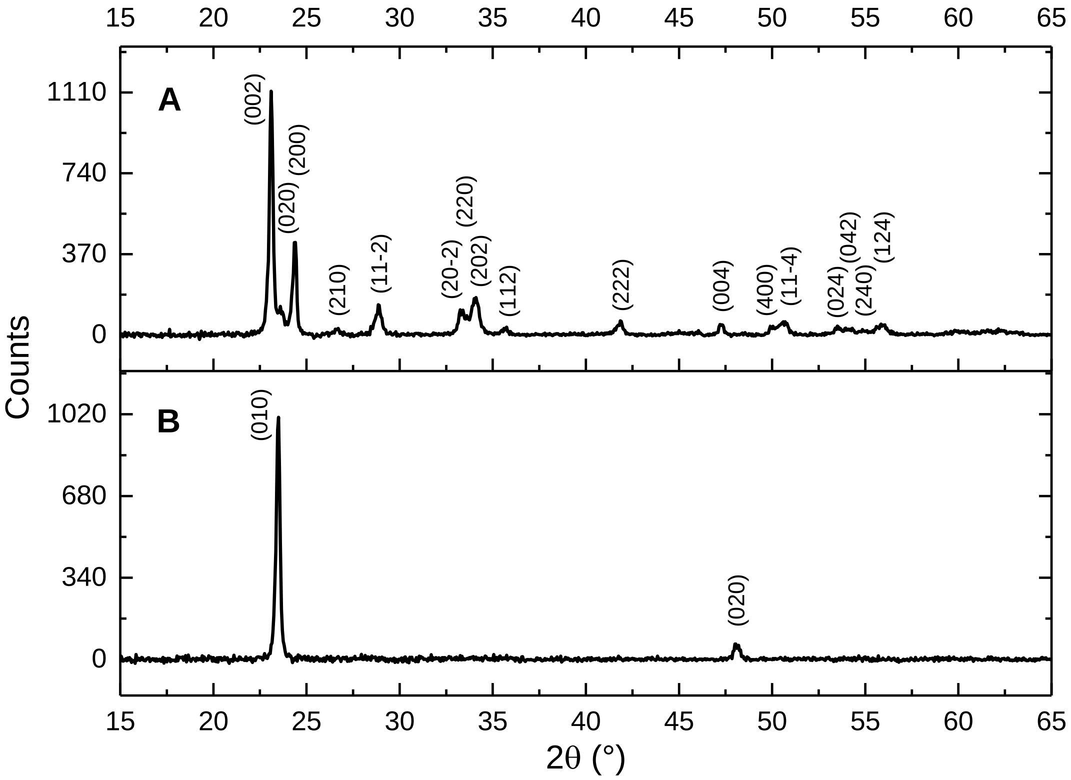

Figure S2. X-ray diffraction patterns of (**A**) m-WO_3 and (**B**) b-WO_3. Labels correspond to the structures ICSD 01-083-0950 and ICSD 01-079-0171 for m-WO_3 and b-WO_3, respectively.

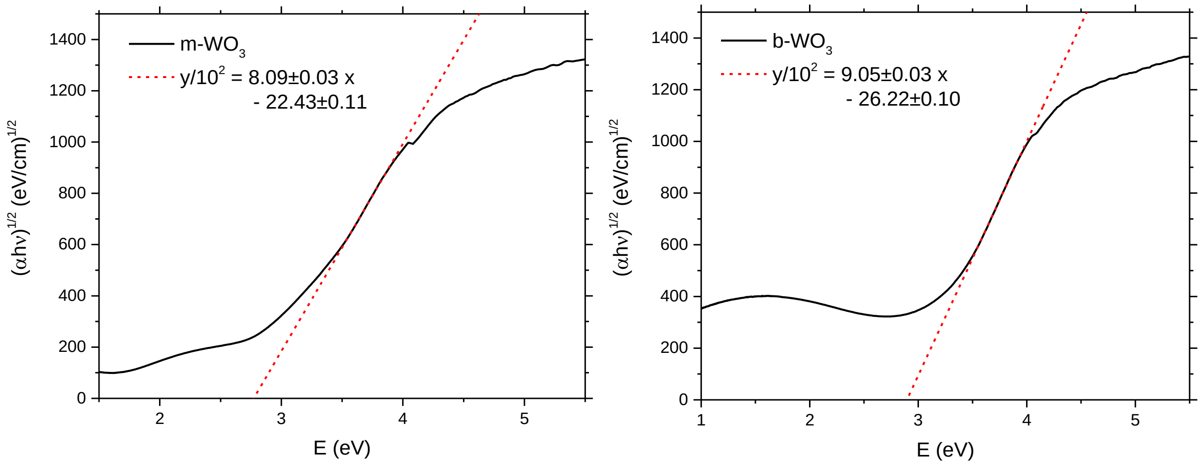

Figure S3. Tauc plots for (left) m-WO_3 and (right) b-WO_3, assuming an indirect band gap for the two materials. Error estimates given in the main paper reflect variations of the extracted band gap over different suitable linear fits.

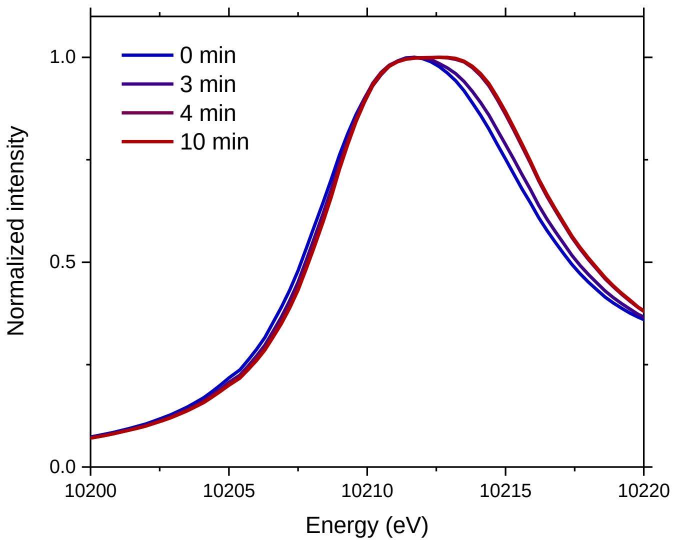

Figure S4. W L_{III}-edge XANES spectra of a separate set of WO_3 samples prepared using a deposition temperature of 300 °C with subsequent annealing times from 0 min (unannealed) to 10 min. The lower deposition temperature of 300 °C compared to the 350 °C used for the m-WO_3 and b-WO_3 samples results in a flatter morphology, thus requiring shorter subsequent annealing times.

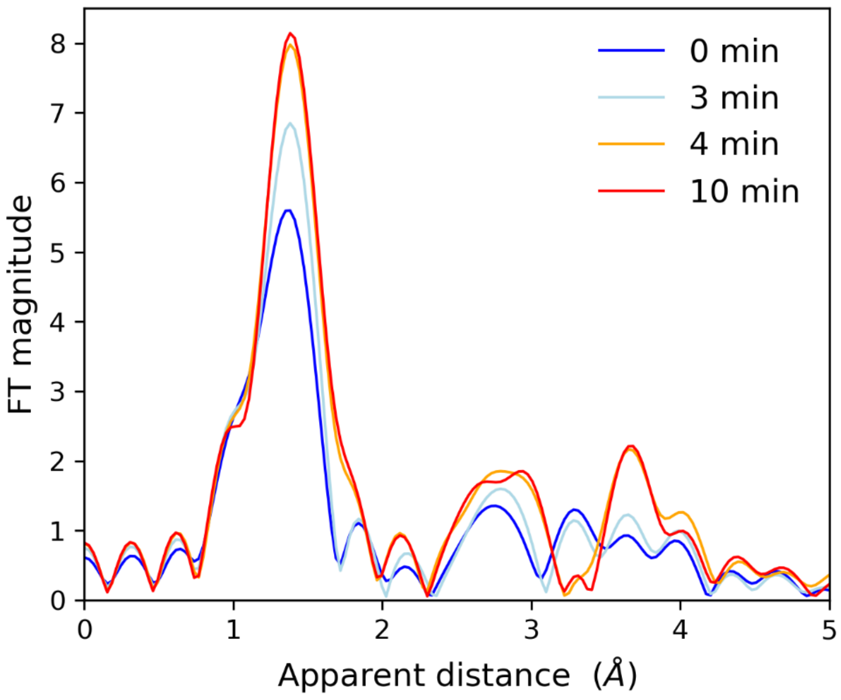

Figure S5. W L_{III}-edge Fourier Transformed EXAFS (k^3-weighted) of a set of WO_3 samples prepared at different annealing times. The extreme cases are: 0 min, corresponding to oxygen deficient WO_3 (b-WO_3) and 10 min, corresponding to oxygen rich WO_3 (m-WO_3).

Figure S5 shows the Fourier Transform K^3-weighed EXAFS spectra of the b-WO_3 (0 min) and m-WO_3 sample (10 min) as well as intermediate states. Comparison of the FT data for the extreme samples reveals a different bonding environment as previously observed for similar samples.[1] According to the literature the different scattering peaks observed are assigned to: (i) 1-2 Å, first shell of oxygen atoms; (ii) 2.3-3.1 Å, multiple scattering within the first shell; this peak is divided in two parts suggesting the existence of two distinctive W-O distances and (iii) 3.1-4 Å W-W distances.[2] As expected fitting of the first shell requires two W-O distances. As shown in **Table S1**, for the near-stoichiometric sample the fits improve upon increasing the W-O distance and a reasonable fit is obtained with a coordination of 4:2, expected for a distorted octahedral. In contrast, for the oxygen deficient b-WO_3 sample (**Table S2**) better fits are obtained with shorter W-O distanced and reasonable results are obtained for different coordination number (2:4, 3:3 and 4:2). This indicates that the sample cannot be described as purely octahedral but that a larger heterogeneity of distances exists. This is in agreement with the lower amplitude observed for this sample indicative of a larger disorder. These results suggest that the generation of oxygen vacancies results in geometric deformations around the W-Centre.

Table S1. EXAFS fit parameters for m-WO_3.

Bond	N	R, Å	σ^2, Å^2	R, %
W-O1	2	1.768±0.017	0.000±0.001	4.3
W-O2	4	2.01±0.04	0.023±0.009	
W-O1	3	1.771±0.012	0.002±0.001	1.62
W-O2	3	2.045±0.019	0.012±0.003	
W-O1	4	1.767±0.013	0.005±0.001	1.2
W-O2	2	2.056±0.014	0.005±0.002	
W-O1	5	1.73±0.05	0.009±0.003	18.9
W-O2	1	2.54±0.11	0.002±0.012	
W-O1	6	1.71±0.06	0.012±0.003	27

Table S2. EXAFS fit parameters for b-WO_3.

Bond	N	R, Å	σ^2, Å^2	R, %
W-O1	2	1.770±0.008	0.004±0.001	0.53
W-O2	4	1.75±0.06	0.021±0.005	
W-O1	3	1.783±0.010	0.006±0.001	0.56
W-O2	3	1.95±0.04	0.024±0.006	
W-O1	4	1.787±0.010	0.008±0.001	0.51
W-O2	2	2.01±0.03	0.020±0.006	
W-O1	5	1.793±0.015	0.009±0.001	0.78
W-O2	1	2.06±0.03	0.009±0.006	
W-O1	6	1.771±0.021	0.013±0.001	3.6

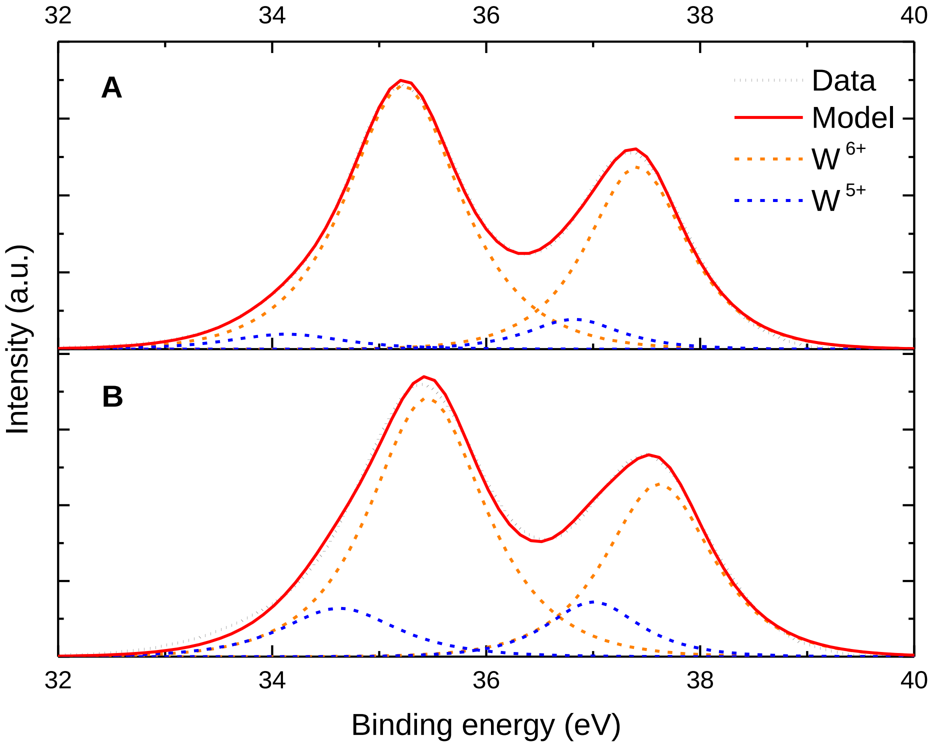

Figure S6. XPS of (**A**) m-WO_3 and (**B**) b-WO_3 in the W binding energy region, reflecting the ratio of W^{5+} to W^{6+} states at the film surface.

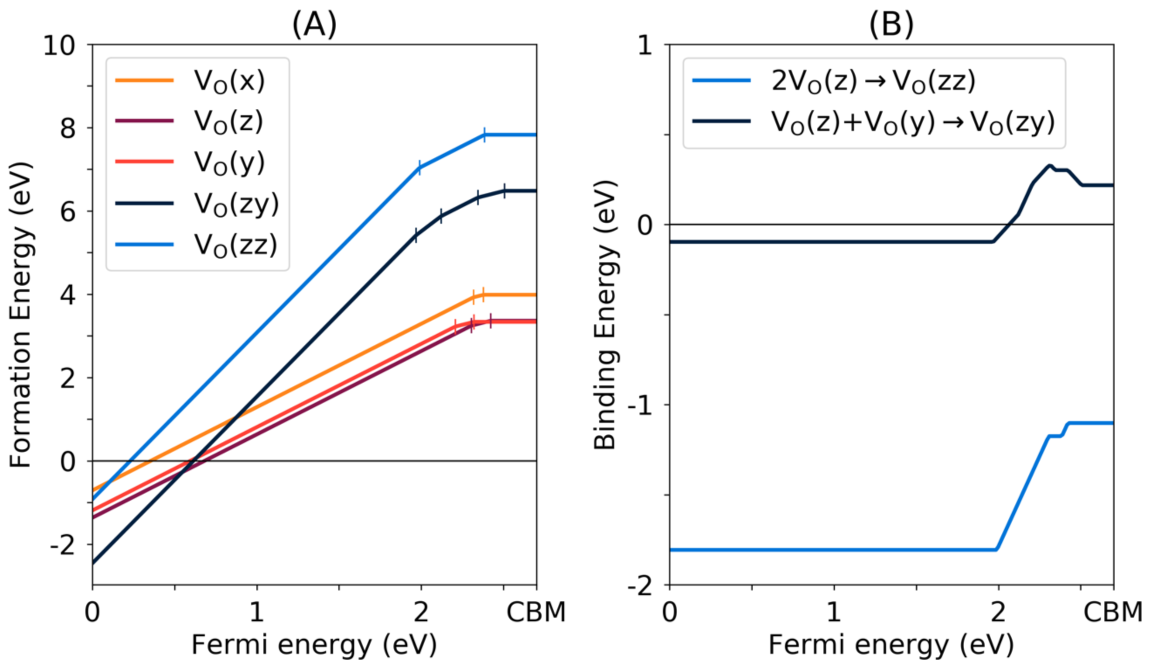

Figure S7. The calculated formation energy of oxygen vacancy defects (**A**) and the binding energy of defect complexes (**B**). In (A), each solid line represents the formation energy of a given defect. We plot only the formation energy of the most stable charge state for a given Fermi energy. The slope represents the charge state (q); the formation energies contain a term qE_F in this grand-canonical formalism. Short vertical lines indicate the thermal transition levels at which the charge state changes. Data in (b) shows how much energy is donated to the ambient system when two oxygen vacancies form a defect complex. This quantity is referred to as binding energy. The binding energy is in general represented as $E_b(A+B;E_F) = E_f(A;E_F) + E_f(B;E_F) - E_f(A+B;E_F)$. In this case, $E_f(A;E_F)$ represent the formation energy of defect A at a given E_F, which can be found in panel (A). Positive binding energy indicate that the defect complex is spontaneously formed.

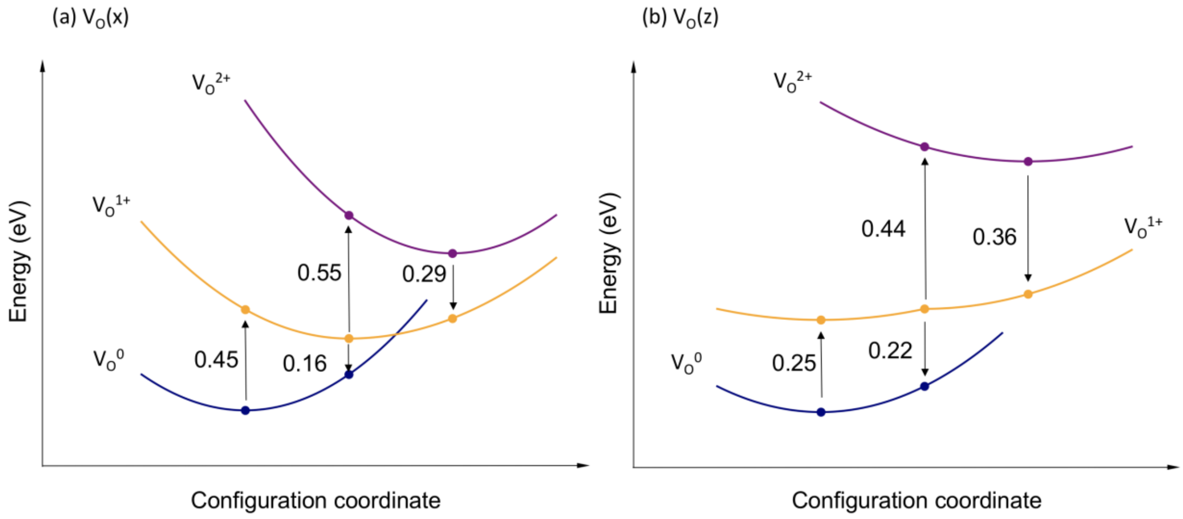

Figure S8. Calculated configuration coordinate diagram of the two oxygen vacancy defects (a) $V_O(x)$ and (b) $V_O(z)$ in three charge states.

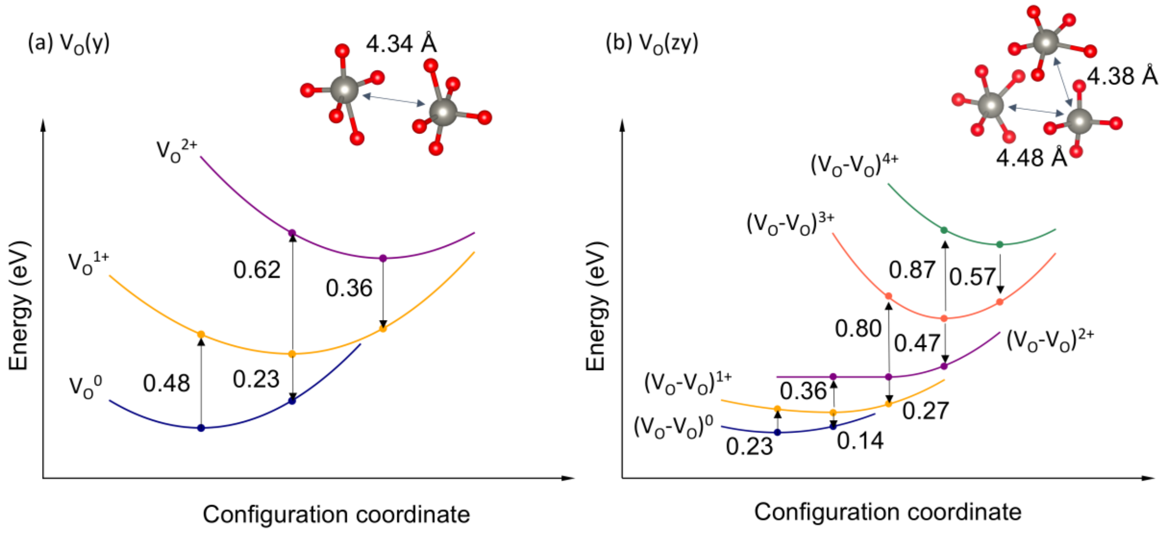

Figure S9. Configuration coordinate diagrams showing the potential energy surface associated with defect ionization, calculated from the density functional theory, for: (a) a single oxygen vacancy and (b) a defect complex composed of two oxygen vacancies. Local atomic structures and the distance between the W atoms are also shown. The V_O^0 to V_O^{1+} and V_O^{1+} to V_O^{2+} transitions are 0.5 eV and 0.2 eV for the isolated vacancy, respectively. Upon complex formation, a wider range of charge states are accessible (four excess electrons), with excitation energies ranging from 0.2 eV to 0.9 eV. n-type conditions are assumed with the Fermi level at the conduction band minimum of WO_3.

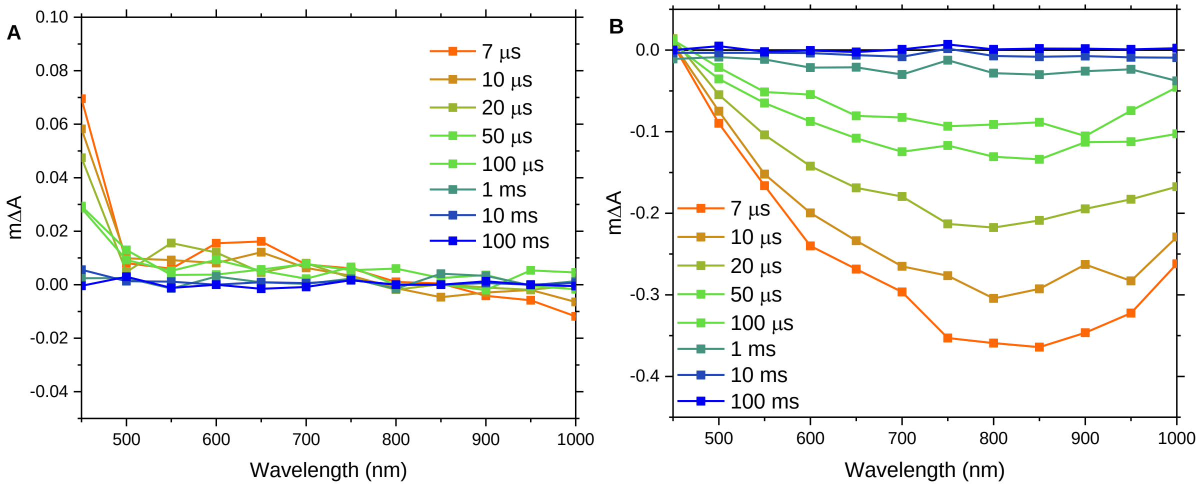

Figure S10. Transient absorption spectra of m-WO_3 (**A**) and b-WO_3 (**B**) for excitation at 355 nm. Spectra were acquired at an excitation intensity of 3.0 mJ/cm^2 under argon atmosphere.

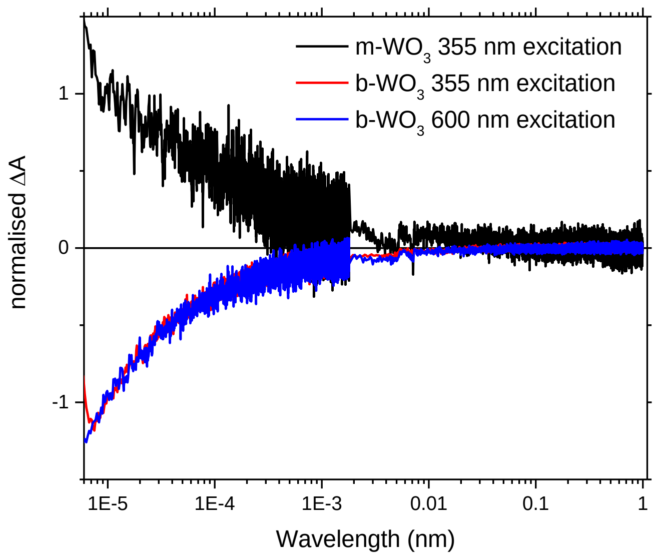

Figure S11. Transient absorption decay kinetics for m-WO_3 at 355 nm excitation and 450 nm probe (black), b-WO_3 at 355 nm excitation and 600 nm probe (red), and b-WO_3 at 600 nm excitation and 650 nm probe (blue). Kinetics were acquired at excitation intensities of 3.0 mJ/cm^2 (355 nm excitation) and 1.5 mJ/cm^2 (600 nm excitation) under argon atmosphere. Samples were excited through the quartz substrate (back excitation).

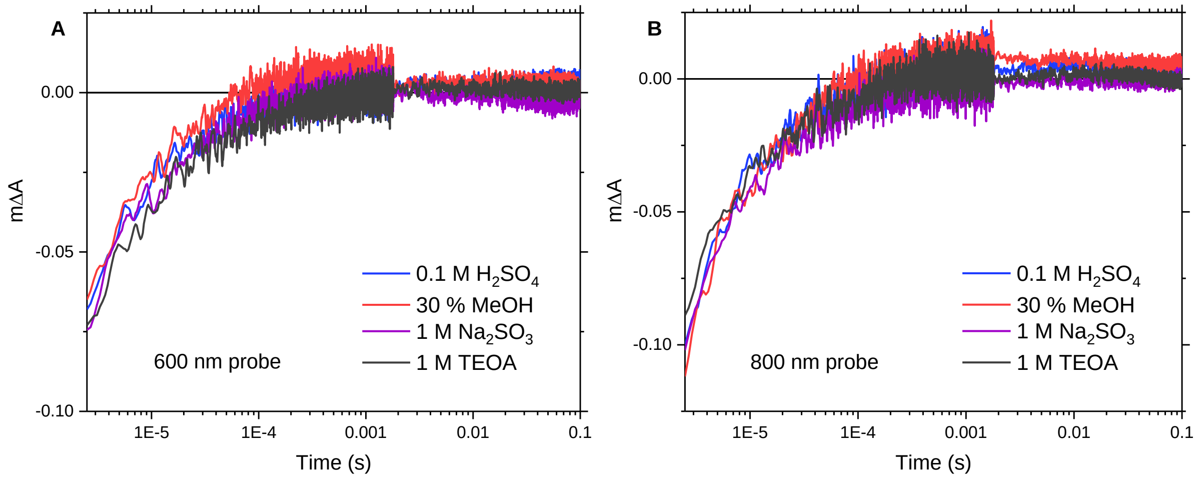

Figure S12. Transient absorption decay kinetics for b-WO_3 at 355 nm excitation for 600 nm probe (**A**), and 800 nm probe (**B**). Kinetics were acquired at an excitation intensity of 1.5 mJ/cm^2 in 0.1 M H_2SO_4 electrolyte only (blue) and after addition of 30 % by volume methanol (red), as well as in the presence of 1 M Na_2SO_3 (purple) and 1 M triethanolamine (black). The sample was excited from the film side (front excitation).

References

1 A. Kuzmin and J. Purans, *J. Phys. Condens. Matter*, 1993, **5**, 2333–2340.

2 T. Pauporté, Y. Soldo-Olivier and R. Faure, *J. Phys. Chem. B*, 2003, **107**, 8861–8867.
